# ‘They just came with the medication dispenser’- a qualitative study of elderly service users’ involvement and welfare technology in public home care services

**DOI:** 10.1186/s12913-021-06243-4

**Published:** 2021-03-19

**Authors:** Heidi Snoen Glomsås, Ingrid Ruud Knutsen, Mariann Fossum, Kristin Halvorsen

**Affiliations:** 1 Faculty of Health Sciences, Institute of Nursing and health promotion, Oslo Metropolitan University, Postbox 4, St. Olavs plass, N-0130 Oslo, Norway; 2 Faculty of Health and Sport Sciences, Department of Health and Nursing Science, University of Agder, Postboks 509, N-4898 Grimstad, Norway

**Keywords:** Aged, Home health care, Technology, Telehealth, E-health, Innovation, Relations, Patient participation, qualitative research

## Abstract

**Background:**

Public home care for the elderly is a key area in relation to improving health care quality. It is an important political goal to increase elderly people’s involvement in their care and in the use of welfare technology.

The aim of this study was to explore elderly service users’ experience of user involvement in the implementation and everyday use of welfare technology in public home care services.

**Method:**

This qualitative study has an explorative and descriptive design. Sixteen interviews of service users were conducted in five different municipalities over a period of six months. The data were analysed using reflexive thematic analysis.

**Results:**

Service users receiving public home care service are not a homogenous group, and the participants had different wishes and needs as regards user involvement and the use of welfare technology. The analysis led to four main themes: 1) diverse preferences as regards user involvement, 2) individual differences as regards information, knowledge and training, 3) feeling safe and getting help, and 4) a wish to stay at home for as long as possible.

**Conclusion:**

The results indicated that user involvement was only to a limited extent an integral part of public home care services. Participants had varying insight into and interest in welfare technology, which was a challenge for user involvement. User involvement must be facilitated and implemented in a gentle way, highlighting autonomy and collaboration, and with the focus on respect, reciprocity and dialogue.

**Supplementary Information:**

The online version contains supplementary material available at 10.1186/s12913-021-06243-4.

## Background

In Western societies, the increasing number of elderly people, financial challenges and early hospital discharges are setting pressure on public home care services [1, 2]. In addition, a shortage of registered nurses and nurse assistants gives rise to problems regarding the quality of health care delivered [3]. It has been a goal during the last two decades to enable the elderly to take care of themselves in their homes for as long as possible [1, 2]. Remaining in a familiar environment is expected to increase independence, is cost-effective and helps the elderly to maintain their health [1, 4]. The implementation of welfare technology in public home care services is a response to the challenge posed by the increasing number of elderly people with care needs [5]. In this study, we rely on one of the most used definitions of welfare technology in Norway:

*‘Technology that can contribute to increased security, safety, social participation, mobility and physical and cultural activity, and that strengthens individuals’ ability to manage for themselves in everyday life despite illness and social, mental or physical disability. Welfare technology can also function as technological support for next of kin and otherwise help to improve accessibility, resource utilisation and the quality of service provision. Welfare technology solutions can in many cases prevent the need for services or hospitalisation’* [6]:99).

Most European countries have also adopted a policy for user involvement and empowerment that creates expectations of quality improvement in public services [1, 2, 7, 8]. Despite increased awareness of user involvement, municipalities struggle to overcome the challenges associated withs translating the rhetoric of involvement into practice [9, 10].

New knowledge of elderly service users’ experiences of involvement in the implementation and use of welfare technology in public home care services is needed and is the purpose of this study.

### Public home care services

Public home care services appear to differ between and within countries. In most countries, they include rehabilitative, therapeutic and assistive home care, in addition to nursing [11]. In Norway, all citizens with health-related needs have a legal right to receive public home care services free of charge [12]. Public home care is organised by geographic area, and it is an integral part of the municipal health care services that are primarily financed through taxes [13]. Although some service users or next of kin acquire welfare technology privately, the most common situation in Norway is that the municipal service acquires, offers and operates the welfare technology, as is the case in this study.

### Welfare technology

In Scandinavian countries, the term welfare technology is commonly used to describe technological solutions used in home care to support and improve services [14]. In the international literature, terms such as telecare, telehealth, telemedicine, assistive living technology and e-health are often used synonymously with welfare technology [15, 16]. There appears to be no consensus on the boundaries between the terms and their content [17, 18].

The goal of using welfare technology is to strengthen individuals’ ability to manage for themselves in everyday life and cope with their life situation, preferably in their own homes [19–21]. Moreover, the use of welfare technology comes with an assumption of increased safety for service users and their next of kin [4, 22]. Welfare technology is expected to contribute to innovation in health care services, with the focus on improved quality and reduced costs [2]. Financial savings associated with the use of welfare technology are primarily related to a reduction in the number of visits from the home care service, fewer hospital admissions and service users being able to stay longer in their homes [23]. There may be discrepancies between expectations and the complex reality these technologies are part of [21]. Previous studies have shown that the introduction of welfare technology can be beneficial, but also have problematic implications and barriers. Not all technology is suitable for service users [4, 24]. Examples of barriers to the implementation and use of welfare technology include attitudes and resistance from users [4, 25], limited knowledge, competence and information [26–28], instability of internet access and the cost of software [26, 29]. These barriers can have an impact on how use is experienced and affect user involvement.

### User involvement

In contemporary Western societies, user involvement is a widely accepted democratic principle, and several countries, including Norway, have developed legislation to strengthen service users’ influence [7, 30]. There are political expectations that user involvement will contribute to increased quality and efficiency, and reduce health care costs [31–33]. However, user involvement is in many ways a vague concept that covers many different approaches [8, 34]. Historically, user involvement is linked to individuals’ right to be able to influence their own lives [35]. User involvement has subsequently been seen as an expression of a consumer and individual orientation, where the focus on freedom of choice is central [8]. In recent years, user involvement has been associated with the terms ‘co-creation’ or ‘co-production’, where users of welfare services are seen as equal and competent co-producers with expertise and a right to influence and improve the services they need [8, 33, 36].

User involvement is about creating opportunities for service users to express their opinions about the service, including sharing information and feelings. The service users’ goals, needs, and capabilities should be the guiding principle for services and interventions if genuine user involvement is to be achieved [33, 37]. For this reason, this study should also be seen in light of the increased focus on patient-centred care in health services [38, 39]. Furthermore, user involvement is also about the relationship between health professionals and service users [40]. Studies and reports have highlighted that service users who are actively engaged in their health and care can experience better health outcomes and care experiences [7, 33, 41]. However, research shows that, in many situations, user involvement is inadequately integrated into health care for elderly [34, 42]. There are several studies from hospitals and discharge processes to home care or mental health, but few empirical studies on user involvement are from public home care services [34, 42–44].

Perceptions of user involvement differ among elderly service users. Some service users believe that user involvement is about receiving information and only have a limited wish to participate in decision-making about the services they receive [45]. Others perceive user involvement as the ability to become involved as co-producers and to be able to decide for themselves [46]. The study by Bjørkquist et al. [5] indicates that involving elderly service users in the process of implementing welfare technology is challenging due to a lack of competence and information about what technology is available and what service users might benefit from. Another study found that elderly service users reported that they often struggled to understand and remember the information they were given about welfare technology, which is a challenge for user involvement [42]. Bennett [19] argues that consideration must be given to patients’ decision-making capability and human rights in connection with user involvement.

Several studies have addressed questions concerning user involvement among the elderly and the use of welfare technology separately, but few studies have looked at public home care from the perspective of service users. We believe that greater insight into factors affecting user involvement in this context will benefit all stakeholders, especially those interested in improving care for elderly service users.

## Method

### Aim and study design

The aim of this study is to explore elderly service users’ experience of user involvement in the implementation and everyday use of welfare technology in public home care services.

A qualitative study with an explorative and descriptive design was chosen. This design offers an opportunity to illuminate experiences and obtain in-depth knowledge of the participants’ experience of user involvement through individual interviews, and to make sense of this knowledge [47]. To explore participants’ experiences, attitudes and reflections on what inhibits and what promotes user involvement, we used a phenomenological-hermeneutical approach [47]. This approach was used in order to capture the essence of the participants’ everyday experiences and to interpret them from a user involvement perspective. By inviting participants with varied backgrounds in terms of gender, age, experience and interest in welfare technology, we gained first-hand insight, knowledge and an understanding of the everyday context and complexity of home health services. This was further strengthened by inviting participants from different municipalities that had taken different approaches to the implementation process, and everyday use of welfare technology.

### Context

Service users of public home care services from five municipalities in Eastern Norway participated. The smallest municipality had approximately 5000 inhabitants, while the largest had approximately 87,500 inhabitants. In terms of land area, the municipalities varied between 176 sq. km and 961 sq. km, and both urban and rural municipalities were included.

The five Norwegian municipalities were obliged to implement and use welfare technology in their day-to-day provision of home care services. The implementation had started, but the municipalities were at different stages of the process in terms of what they were able to offer their inhabitants.

### Recruitment and participants

During planning of the data collection, a joint information meeting was held for the management of some of the municipalities that Oslo Metropolitan University had a cooperation agreement with. They were informed about the objective and the planned research design of the study. Managers of home care services in three municipalities accepted the invitation to participate. To ensure enough participants, two more municipalities were invited.

The inclusion criteria for taking part in the present study were that the service users were capable of giving consent, had used some kind of welfare technology for at least six months, were 65 years old or older, and able to sign an informed consent. It was requested that participants have varied backgrounds in terms of gender, age, experience of and interest in welfare technology.

The management of home care services asked the health professionals who were in daily contact with service users to give potential participants an information form containing information about the study and the written informed consent that was to be signed. As soon as the home care service received the written consent and delivered it to the first author, the participants were contacted by phone, and interviews were scheduled. Initially, 18 participants consented to take part in an interview, but two withdrew before the interviews took place.

A total of 16 participants, five men and 11 women, ranging in age from 65 to 95 years, participated in the study. Some had used welfare technology, such as safety alarms, for a few months and others for many years, while a few respondents did not remember exactly how long they had used welfare technology. Digital safety alarms, medication dispensers and digital door locks were the most used welfare technologies in these municipalities.

### Data collection

A semi-structured interview guide was developed by the authors for this study (Additional File 1). The interview guide was designed to explore participants’ experiences systematically and comprehensively, and it kept the interviews focused on the desired line of action. Nonetheless, as Bowling [47] recommends, it allowed the interviewer to probe and enabled the participants to raise other relevant issues. The questions in the interview guide comprised core questions and many associated questions, which, in turn, were further improved through one pilot test in line with Cresswell’s recommendations [48]. The main questions in the interview guide were whether participants could tell the interviewer about: how they obtained the technology, how they used it, whether, in their experience, health professionals from the municipal health services were attentive to their needs and wishes, whether they experienced being involved in processes, and what challenges or needs they believed had to be addressed to ensure more user involvement. The interviews took place between March and September 2019.

The first author conducted the individual interviews and met the participants for the first time at the interviews. The first author assessed the participants’ competence to consent and decided whether it was appropriate to conduct the interviews. Only the participants and the first author were present during the interviews. The first author is an RN/PhD student and has previous experience of individual interviews and qualitative methods. In the interviews, it was desirable to be attentive to the participants’ experiences and stories and to be sensitive to surprises, topics and opinions that might challenge preconceptions. A few participants had some problems expressing themselves during the interviews, after e.g. stroke or mild cognitive impairment. This meant that it was necessary to make adjustments during some of the interviews in order to specify and explain some questions. Simple verbal prompts were provided to improve the communication. Moreover, some answers were very brief, with the result that some of the data were of low quality, while others provided rich data. Both during and after the interviews, the first author took notes for the analysis. When the 16 interviews were completed and a preliminary analysis had been carried out, the authors agreed that satisfactory saturation had been achieved.

Three interviews took place at a day activity centre for the elderly, and the rest in the participants’ homes. The interviews lasted between 30 and 90 min; they were recorded digitally and transcribed verbatim and unidentified. The first author transcribed eight of the interviews, and a professional transcriber the rest.

### Data analysis

The coding in the data analysis was performed using NVivo 12 software. After the coding, manual analyses were carried out. All authors were involved in the analyses.

Reflexive thematic analysis was used, as described by Braun et al. [49]. In the first phase, the objective was familiarisation with the data. All the interviews were read and reread by all the authors, and possible interpretations of the material were discussed. In phase two, more detailed and systematic work was carried out. We extracted the meaning content from the data and generated codes, using open thematic coding for each transcript. The NVivo12 software provided an overview and helped us to organise and manage the data in the process. In phase three, we grouped codes and manually constructed initial themes. Themes were identified and discussed across the data and in line with the research questions and our interpretations. Some statements were categorised under more than one theme. In phase four, we revised initial themes and discussed themes back and forth to avoid overlaps. We discussed how the themes were related to each other across the whole data set. In phase five, the themes were revised and given more clarified names that conveyed their essence. It was a goal that the final themes should reflect the results. The analytical work was wrapped up in the sixth phase, which involved checking how well the themes worked, together and individually, and preparing the article. Throughout the analysis, the authors went back and forth in the data material.

The results were presented to an external project advisory group for the PhD project of which this study is a part. Participants in this group were recruited from two Pensioners Associations, and one from a next of kin group of the National Association for Public Health. The group consisted of one person receiving home care and two next of kin, one of whom was also a retired nurse assistant. The participants in this group acted as discussion partners in the interpretation of the findings. The responses did not produce any immediate changes but confirmed that the analytical reflections were in line with their experience.

The Consolidated Criteria for Reporting Qualitative research (COREQ) checklist for reporting qualitative studies was used [50].

### Research ethics

The Helsinki Declaration’s principles for medical research [51] were complied with. All respondents in the study were given oral and written information about the project and signed a written informed consent. Information was also provided about the possibility of withdrawing from the study if they wished before the data were analysed. Since the participants were a vulnerable group of frail elderly, competence to consent was assessed before the interviews were conducted. All data were anonymised, and the confidentiality of the respondents was safeguarded. The data were stored in accordance with the applicable rules and guidelines for storing research material. The project was approved by the Norwegian Centre for Research Data (NSD), reference number 473910.

## Results

The participants had varied backgrounds as regards their health, social and economic status, and they had different needs and experiences. Most of them lived alone, and there were significant variations in housing standards. While some lived relatively isolated, far from their nearest neighbour and in simple living conditions, others had moved to modern, practical apartments or to an independent living facility with services in the city centre area. Some of the participants had problems remembering or expressing themselves orally, for example after having had a stroke. In contrast, others had no problem at all and eagerly kept up with the news and were socially engaged. Some participants expressed that they were active users of Skype, Facebook and other types of social media, while others were not on the internet and had neither a mobile phone nor a computer.

Four main themes emerged during the analysis. There were challenges related to involvement in the decision-making process because of the different preferences among the participants as regards user involvement. Moreover, individual differences in information, knowledge and training affected the participants’ ability to ask for welfare technology and become involved in the decision-making process. A third theme concerned how the experience of safety affected attitudes to and the use of welfare technology. Participants’ experience of welfare technology as a tool that could enable them to stay as long as possible in the home was the final main theme.

### Diverse preferences for user involvement

The results showed differences between the participants as regards to what extent and in what way they wanted to participate and be involved when welfare technology was implemented and used. Some participants’ attitude was that health professionals knew best and made the correct choices on their behalf when the municipality acquired welfare technology. Other participants said that they did not want to decide because they lacked energy or knowledge. They expressed gratitude for the help they received and said that other service users should also be grateful and not complain or argue when health professionals came up with ideas for new technology. It was a challenge that some of the participants did not remember whether they had been asked if they wished to use welfare technology.

*‘Oh, no, I don’t want to decide. I don’t have enough energy, so the health professionals must choose (the type of welfare technology).’*

Some of the participants felt that they coped well with everyday life themselves, but reported that their family or health professionals argued that they needed to use welfare technology. In some situations, participants felt that others decided for them. For some, this was okay, while, for others, it was not because they wished to make their own decisions. Nonetheless, most of the participants said that they accepted what the health professionals and next of kin thought they needed because they did not have enough knowledge or did not want any conflicts.

*‘No, that’s... it’s the family. They want me to have such a safety alarm, but I do not think I need it.’*

On several other occasions, participants stated that they wanted to be involved when health professionals suggested or brought welfare technology with them. They wanted to discuss the available opportunities with the health professionals and be given a chance to accept or reject the new technology. To be able to make individual choices and having the feeling of being in charge of their own lives were perceived as important.

*‘..they just came with it (medication dispenser). They are a bit.. what can I say ... they are a bit controlling. It would have been nice if they had asked.’*

In procurement processes for welfare technology, the municipalities frequently bought or rented just one model to cover the services in their municipality, and the service users were not invited to participate in the process. The result indicates that the technology was not tailored to the individual service users’ health challenges. In some cases, the result was that some participants could not use the model offered by the municipality, at least not in the expected way. An example from this study was a new type of safety alarm. Several of the participants found it too heavy to wear around their necks. For that reason, they put the safety alarm in their handbag, laid it on the table or hung it on their walking frame. The participants did not reflect on the risk of being unable to access the alarm if they were in need of help. This example also indicates that not all development is necessarily positive.

*‘... it doesn’t work with me. I never wear it on me. I can’t wear anything heavy around my neck. I am very sore in all my muscles and body ... in my skin.’*

### Individual preconditions for knowledge, information and training

Participants’ prior knowledge of welfare technology varied from not knowing what the term meant to having a good overview of what it is, what kind of welfare technology exists and what the municipality can offer. For most of the participants, it was important to be given information about welfare technology in general, and what the municipality could offer in particular.

*‘We need more information because there is a lot that I do not know. What you can apply for, what you are entitled to, such important things.’*

Other participants said that they did not need such information now, but that, if they got worse and some technology could help them in their everyday lives, they would like to be given such information. Based on the results, it also seems that what is known and what is unknown about welfare technology influenced whether service users themselves take the initiative to apply for it. For example, most of the participants stated that they or their next of kin had applied for safety alarms. As regards medication dispensers and digital door locks these were aids that health professionals suggested when they thought it would help service users to cope with everyday life and continue to live at home.

Only to a limited extent did health professionals ask the participants about how they experienced using the technology and what knowledge they felt was lacking. Most participants were satisfied with the use of welfare technology and felt that it was easy to use, even though several of them had only been given limited training. Our results indicate, however, that, in some situations, participants had a limited understanding of how to use the equipment, which may have led to incorrect use.

*‘Because I didn’t know how to ... Because I thought it was just a case of pressing the button, but it was not. You have to touch it and hold it for a few seconds or so. Then you have contact with the home care (safety alarm).’*

### Feeling safe and getting help

The participants expressed that the use of several welfare technologies gave them an experience of safety, and that this was essential if they were to have a positive attitude to using such technology. For example, using safety alarms made the participants feel safer, and this feeling was further enhanced for those who had safety alarms with Global Positioning System (GPS) tracking. None of the participants experienced GPS tracking as intrusive monitoring, only as providing increased safety. This indicates that participants were more concerned about getting help than about the possibility of being monitored. Another positive aspect emphasised by the participants was that if they forgot to charge for example the safety alarm, the health care professionals were notified digitally about the low battery. In such case, the health professionals contacted the participants and asked them to charge it. The home care service was also notified if the service user forgot to take the medication from the medication dispenser, or if there was something wrong with the dispenser, for example, if the medication inside the dispenser had jammed.

*‘It is the safety that makes it okay to have one (safety alarm), so you can get hold of someone if you should fall.’*

The results indicated that, for some participants, the conditions for involvement and understanding information were challenging. It emerged from our findings that some participants’ insight into their own cognitive capacity was limited. For example, some of them stated that they did not understand why the medication dispenser repeated that they had to take their medicine. They said they sometimes became irritated and thought that the dispenser was being ‘fussy’. Experiences also differed as regards whether health professionals observed that they mastered the use of welfare technology. Most of the participants said that it was reassuring that the health professionals checked how they used it, while, for others, it was perceived as rather controlling and indicated a lack of confidence in them.

*‘Now, they do not check. In the beginning, they did, but then they realised that I could remember how to do it myself.’*

Some of the participants had experienced some start-up problems with the technology, for example related to an unstable network or software issues. When the welfare technology had faults or did not work, this could represent a safety risk for the service users, for example if the health professionals could not open the digital door lock when service users were in need of help. The participants did not emphasise such challenges. Instead, they found pragmatic approaches until the problem was solved. For example, participant 18 stated: *‘It still happens a few times that the door lock does not work, but I keep the balcony door open and the health professionals can enter by it, so it is not a problem.’* The participants did not perceive an unlocked balcony door as a security problem and seemed to be less afraid of uninvited guests than of not getting help when needed.

### A wish to stay at home as long as possible

Several of the participants stated that welfare technology was a prerequisite for continuing to stay at home. They were positive about making more use of welfare technology if that would enable them to cope with their everyday. When they experienced, for example, that technology helped them to remember to take their medicine, this gave them a feeling of mastery.

*‘I think you can say that everyone should try a medication dispenser. They will become so fond of them. In the morning when I get up, the dispenser says, “it’s time for medicine” and then I manage to take it myself.’*

None of the participants reported that they had been asked whether they would prefer a visit from a health professional to administer their medication instead of using the medication dispenser. However, most participants said that they preferred the medication dispenser. One argument that was mentioned several times in the interviews was the importance of taking medication at the right time, instead of waiting for the health professionals to come, which had previously been a problem. Managing the administration of medication gave the participants a feeling of independence and increased freedom, which illustrates that, for some service users, technology can be experienced as better than the services provided by health professionals.

*‘The home care service came up with this idea, and I thought it was a gift package (medication dispenser). It is a lot easier because, if people come here every day, then I have to ... then I am very tied up.’*

However, some participants preferred physical visits for the administration of medication but were not given this option. For them, health professionals represented social contact in a situation where they struggled with loneliness.

*‘If I had the choice, I would have chosen someone to come. I am alone a lot. I think it is nice when someone comes here and talks to me.’*

## Discussion

When we started the study, our objective was to explore participants’ experience of involvement in the implementation and everyday use of welfare technology in public home care services. After our analysis, the results showed that we could not refer to elderly people living at home as a homogeneous group. They are a group of individuals with very different knowledge, needs and preferences as regards user involvement and how to use welfare technology.

The results illustrate various aspects of involvement, engagement and dialogue about the implementation and use of welfare technology. Based on the results from this study, we would argue that user involvement seems to be more of an ideal than normal practice in home care services, even though it has been a legal requirement and a political goal for some time [1, 2, 7, 8].

### Conditions and challenges for involvement and decision making

The results, which are in line with other studies [34, 37], show that some participants felt that they were not involved, which may lead to a feeling of disempowerment and resignation. This can especially be the case if the service user gets the feeling that health professionals have made up their minds before discussing with them, which is supported by the study by Rydeman and Törnkvist [52]. Health professionals’ attitudes and whether they focus on users’ needs and goals in their contact with service users may have an impact on the service users’ feeling of involvement. Hestevik et al. [53] argue that a paternalistic attitude on the part of health professionals in relation to how service users are included in the process and allowed to share their wishes and experiences can be a barrier to user involvement. In line with Olsen et al. [39], a more patient-centred focus can contribute to user involvement in relation to service users’ health-related needs and goals.

The study revealed differences between the participants as regards to what extent and in what way they wanted to be involved in decision making, which other studies also support [34, 54]. It is pertinent to ask what is realistic to expect of elderly service users, especially in relation to see them as equal and competent partners, as in co-production, since many of them have multimorbidity and experience low energy. As Bennet [19] pointed out, it is necessary to consider the consequences of inviting frail service users to be more involved, and whether this could lead to a feeling of negative mastery. Our results indicate that involvement and democratic ideals may be overwhelming and too much to expect from frail service users, as also Paillaud et al. found [55]. In line with our findings and Pearson et al. (2015), service users sometimes want health professionals or their next of kin to make choices for them. This indicates that service users have trust in health professionals, but it could also be because many elderly service users are accustomed to the traditional, paternalistic and task-oriented care approach [34, 53, 56, 57]. However, when service users do not want to be involved, their autonomy should be respected. Choosing not to be involved can also be seen as a form of user involvement.

Cozza et al. [58] point out that welfare technology works differently in different contexts and for different people, something our results also indicate. The material attributes of technologies, such as shape, colour, durability and size, can influence whether and how the technologies are used [18]. Technologies that are meant to be beneficial and to enhance safety might not be suitable if the service user does not use them as intended, as was the case for some of the use of safety alarms in this study, and as also found in the study by Stokke [22]. This highlights the need for user involvement by end-users in the procurement process if the home care service is to acquire new technology that actually meets service users’ needs. Furthermore, other studies [4, 39] point out that health professionals have a responsibility to follow-up on what service users experience as important.

Health care decision-making is complex and requires efficient and explicit processes to ensure transparency and consistency of criteria considered [59]. Health decision-making frameworks provide policymakers with evidence to inform decision-making [60]. Weights on criteria in frameworks vary widely, reflecting the diverse perspectives of involved participants [61]. In this process, service users’ involvement and reflections about social, economic, organisational, and ethical criteria can enrich the framework**.** In situations where information and involvement from frail service users can be challenging, alternative data collection should be considered. If data from one essential group is missing, decisions of criteria can be made on an inadequate basis, and there is a risk that quality work will fail.

### The need for knowledge, information and training

Knowledge, information and training is a prerequisite for exercising the right to be involved, and it must be adapted to the individual’s needs, as enshrined in the Norwegian Patients’ Rights Act [30]. Our study found shortcomings in this area, and we also found that limited knowledge and training led to uncertainty and resistance to the use of welfare technology, which is in line with the study by Nilsen et al. [25]. Sufficient knowledge and information are also necessary for service users to be able to look after their health, self-manage their own lives and provide input that can increase the quality of the services [22, 33, 62–64]. Our results show that the participants asked for technologies they were familiar with, such as safety alarms. Safety alarms have been well established and much used since the late 1970s in health care services in Western societies [22]. For the other welfare technologies, such as medication dispensers, it was the health professionals who suggested using them. This highlights the need to improve information about what exists and how to obtain it. However, information and communication do not automatically enable service users to influence decisions about the introduction of welfare technology in home care services.

### Welfare technologies’ impact and consequences for safety, independence and the ability to stay at home

Overall, the participants in our study were positive to welfare technology and wanted to use it more because of the feeling of safety it gave them and because it could help them to continue to live an independent life at home, as supported by findings from other studies [4, 19, 20]. For example use of safety alarms enabled participants to keep doing daily activities without worrying about falling, which is in line with a finding from a study by Stokke [22]. Another point of agreement with other studies [21, 65], was the initial scepticism we found about using the technology. After a while, participants felt that welfare technology contributed to their feeling of safety. Such initial scepticism highlights the need for friendly nudging when welfare technology is introduced, and for close cooperation and follow-up to keep users feeling safe.

One consequence of using welfare technology was a reduction in the number of physical visits by health professionals to some participants. Even though this is a desired development from the authorities and some service users’ perspective. Bennet [19] points out that it is essential to consider the impact such changes have on service users. As the results from several studies indicate, it is important to acknowledge that technology in elderly care cannot be seen as a neutral tool, and it is essential to consider the impact its use has on service users [19, 22, 66]. For example, one of the participants was happy about using a medication dispenser but said that, if she had the choice, she would still prefer to have a person come with the medication, because of loneliness. Reduced visits can also be challenging for health professionals as regards identifying whether, for example, a service user’s cognitive function is decreasing, and evaluating whether service users can no longer handle the technology, as pointed out in another study [67].

In some studies, health professionals have expressed some reservations about the impact on civil rights of using GPS tracking [26, 68]. The findings from our study show that the participants did not experience the use of GPS as monitoring, but as a safety measure. What service users see as important should be the guiding principle for services and interventions if genuine user involvement is to be achieved, rather than the attitude of the health professionals, as also supported by other studies [33, 39, 56]. A feeling of being heard and listened to was seen as important by the participants in our study. In line with Kuipers et al. [38], we found that user involvement with the focus on patient-centred care and co-creation of care can have a positive effect on service users’ well-being and satisfaction. Olsen el al [39]. pointed out that patient-centred care is essential for trust and cooperation, as well as for optimising health care. Making patient-centred user involvement the standard way of working among healthcare professionals is a matter of urgency in relation to putting the service user in focus. If this is not done, there is a risk that user involvement will end up as mere rhetoric and not a realistic approach for the public home care service.

### Limitations of the study

This study is based on a sample of 16 participants and the welfare technologies they use, which means that it has a somewhat limited knowledge base. The municipalities were at different stages of implementation which may have affected participants’ experiences and reflections. The participants were frail elderly, which may have had an impact on the responses we received. Nonetheless, the results highlighted challenges for user involvement and everyday use of welfare technology. The authors’ preconceptions and experience could also have influenced the results. To ensure credibility through ongoing reflexivity, all steps in the analysis were discussed with all the authors and tentatively presented in a clear manner.

### Relevance to clinical practice

This study provides valuable knowledge and will increase awareness of different needs and preferences for user involvement among elderly service users. It also highlights the importance of individual assessments, and adds to our knowledge and understanding of the complex nature of public home care. The results show that information, knowledge and training must be facilitated, preferably in a more patient-centred way, if user involvement is to become a reality. The participants were not concerned about monitoring, but about feeling safe, getting help when they needed it, and staying at home for as long as possible. The municipalities must keep this in mind in their further implementation of welfare technology. To further improve the quality of home care services, user involvement should be facilitated and implemented in a gentle and patient-centred way, where the focus is on autonomy and collaboration, as well as on respect, reciprocity and dialogue about the service users’ situation.

## Conclusion

The results indicate that user involvement is only to a limited extent an integral part of public home care services for the service users. Most of the participants called for more knowledge, information, training and opportunities to play an active part in decisions on the use of welfare technology. However, some of them preferred health professionals taking decisions on their behalf. Service users’ autonomy should be respected even when they do not want to be involved. Standard offers of welfare technology and limited dialogue between the home care services and the participants result in limited opportunities for individual adaptation. In some cases, this led to non-optimal use of the technology. Nevertheless, the welfare technology that was already introduced made participants’ feel safer and enabled them to continue to live at home, which was very important and in accordance with political recommendations and goals.

As regards user involvement, our results highlight the challenges of involving frail elderly in the implementation and everyday use of welfare technology in public home care services. A relevant topic for future research would be to look more closely at interventions that can encourage patient-centred user involvement and test it, for example, in a randomised controlled trial.

## Supplementary Information

**Additional file 1:.** User involvement in the introduction and daily use of welfare technology in home care services. Interview guide.

## Data Availability

The dataset is in Norwegian, and the approval from NSD and the participants is only linked to this study. The corresponding author may be contacted on request for access to the dataset.

## Abbreviations

COREQ checklist: The Consolidated Criteria for Reporting Qualitative research checklist.
RN: Registered nurse.
PhD project: Doctoral project.
PhD student: Doctoral student.
NSD: The Norwegian Centre for Research Data
REK: The Norwegian Regional Committees for Medical and Health Research
