# Supplementary material for: ‘They just came with the medication dispenser’- a qualitative study of elderly service users’ involvement and welfare technology in public home care services
Source: BMC Health Serv Res. 2021 Mar 19;21:245. doi: 10.1186/s12913-021-06243-4 (PMC7977566; doi:10.1186/s12913-021-06243-4)
Supplement: Supplementary file 1 — Additional file 1:. User involvement in the introduction and daily use of welfare technology in home care services. Interview guide. [file 12913_2021_6243_MOESM1_ESM.docx]

# User involvement in the introduction and daily use of welfare technology in home care services

**Demographic data**

Age:

Sex:

What kind of welfare technology do you use?

How long have you used this type of welfare technology?

What was the main reason for introduction of the welfare technology?

**Themes**

1: Please tell me about how you obtained……(use here the welfare technology they initially mentioned, e.g. medicine dispenser), and did you feel involved in the process?

2: Can you tell me about your experience of using ……(use here the welfare technology they mentioned) in everyday life, have it changed your life in any way and in that case, what are these changes?

3: Can you tell me about your experiences concerning whether the health care professionals coming from the home care service are interested in listening to your experiences and wishes about the use of …(use the welfare technology they mentioned).

4: Can you tell me how you experience information, guidance and follow-up care from the home care service in relation to the use of welfare technology?

5: Do you have any concerns and/-or worries of using ……(use here the technology they mentioned)

6: Can you tell me how you think user involvement should be further facilitated and implemented to improve of the quality of home care services?

Do you have any other thoughts about user involvement and welfare technology that you would like to share with me?
